# Metabolic Engineering of *Pediococcus acidilactici* BD16 for Heterologous Expression of Synthetic *alaD* Gene Cassette and L-Alanine Production in the Recombinant Strain Using Fed-Batch Fermentation

**DOI:** 10.3390/foods10081964

**Published:** 2021-08-23

**Authors:** Anshula Sharma, Masafumi Noda, Masanori Sugiyama, Baljinder Kaur, Ajaz Ahmad

**Affiliations:** 1 Systems Biology Laboratory, Department of Biotechnology, Punjabi University, Patiala 147002, India; anshula_rs17@pbi.ac.in; 2 Graduate School of Biomedical & Health Sciences, Hiroshima University, Minami-ku, Hiroshima 734-8551, Japan; bel@hiroshima-u.ac.jp (M.N.); sugi@hiroshima-u.ac.jp (M.S.); 3 Department of Clinical Pharmacy, College of Pharmacy, King Saud University, Riyadh 11451, Saudi Arabia

**Keywords:** amino acid production, bioprocess development, fed-batch fermentation, lactic acid bacteria, L-alanine, metabolic engineering, synthetic gene designing

## Abstract

Metabolic engineering substantially aims at the development of more efficient, robust and industrially competitive microbial strains for the potential applications in food, fermentation and pharmaceutical industries. An efficient lab scale bioprocess was developed for high level fermentative production of L-alanine using metabolically engineered *Pediococcus acidilactici* BD16 (*alaD*^+^). Computational biology tools assisted the designing of a synthetic *alaD* gene cassette, which was further cloned in shuttle vector pLES003 and expressed using an auto-inducible P289 promoter. Further, L-alanine production in the recombinant *P. acidilactici* BD16 *(alaD^+^)* strain was carried out using fed-batch fermentation under oxygen depression conditions, which significantly enhanced L-alanine levels. The recombinant strain expressing the synthetic *alaD* gene produced 229.12 g/L of L-alanine after 42 h of fed-batch fermentation, which is the second highest microbial L-alanine titer reported so far. After extraction and crystallization, 95% crystal L-alanine (217.54 g/L) was recovered from the culture broth with an enantiomeric purity of 97%. The developed bioprocess using recombinant *P. acidilactici* BD16 *(alaD^+^)* is suggested as the best alternative to chemical-based commercial synthesis of L-alanine for potential industrial applications.

## 1. Introduction

The global market value of amino acids, especially the nonessential ones, has enhanced significantly over the years owing to their humungous industrial applications [1]. L-Alanine, though a non-essential amino acid, has extensive food, pharmaceutical and veterinary applications [2,3]. It is an FDA-approved food additive and nutritional supplement, mainly used as a low-calorie sweetener, a fat substitute and as an ingredient of therapeutic formulations for treating hypoglycemia, liver diseases, prostate hypertrophy and urea cycle disorders [4,5,6]. It is widely used as a hair and skin conditioning agent and as an ingredient in cosmetics and several personal care products [7,8]. Additionally, it has also been explored as feedstock for the manufacture of engineered thermoplastics [9].

Commercially, L-alanine production is achieved by enzymatic conversion of L-aspartic acid using immobilized cell suspensions of *Pseudomonas dacunhae* [10], which is limited by factors such as high production cost and poor substrate availability, as the substrate L-aspartic acid is usually produced from fumaric acid, which is further obtained from the non-renewable petroleum feedstock [11]. Thus, to meet the increasing demand, biotechnological approaches offer a great alternative to chemical synthesis for the cost-effective production of L-alanine using a “biological” way. The most preferred route for microbial L-alanine synthesis from pyruvate via glycolytic pathway is facilitated by the alanine dehydrogenase enzyme (AlaDH), which catalyzes the reductive amination of pyruvate into L-alanine. L-alanine production from pyruvate by NADH-linked AlaDH has already been reported in various microorganisms, such as *Arthrobacter oxydans* [12], *Bacillus sphaericus* [13], Clostridium sp. P2 [14], recombinant *Corynebacterium glutamicum* [15,16], recombinant *Escherichia coli* [11,17], recombinant *Lactococcus lactis* [2] and *Zymomonas mobilis* [18]. However, the production of L-alanine in most of the previously reported native and recombinant microbial strains suffered from slow fermentation productivities, lesser purity and poor yield, due to the formation of racemic mixtures and co-products.

L-alanine is mainly used in the food industry; thus, the functional starter culture lactic acid bacterial strains could serve as potential hosts for microbial L-alanine production. However, till date, no starter culture bacterium (except for recombinant *Lactococcus lactis*) has been explored for the fermentative production of L-alanine at a commercial scale [2]. These limitations have aroused a dire need to develop an efficient bioprocess for the microbial production of L-alanine by employing synthetic metabolic engineering strategies for strain improvement and using efficient fermentation approaches such as fed-batch fermentation for enhancing production yields.

Fed-batch fermentation has been widely employed for the enhanced production of desirable microbial metabolites. The fed-batch cultivation process is being carried out by intermittent or continuous addition of essential nutrients to the culture vessel for optimizing cell growth and maximization of product yield during fermentation [19]. In fed-batch cultivation processes, feeding of a substrate or carbon source at defined time intervals and concentrations is one of the most important factors for efficient conversion of substrate into the desired end product. However, the substrate additions should be carefully monitored to avoid any inhibitory effect. Despite the above limitation, the fed-batch cultivation process offers several advantages, such as improved cell biomass, enhanced productivity, reduced substrate or end-product inhibition, reduced viscosity of the culture broth and elevated dissolved oxygen rate [20].

Metabolic engineering strategies in combination with synthetic biology approaches intend to enhance the production of desirable metabolites in the industrially relevant microorganisms by tailoring their genetic and regulatory processes [21,22,23,24]. Therefore, the present study was designed to tailor existing cellular metabolism of *P. acidilactici* BD16 for the construction of a novel L-alanine production pathway, which can boost its potential industrial applications. The Gram-positive homo-fermentative lactic acid bacterial strains have been extensively harnessed for various food-based applications due to their probiotic and GRAS (generally recognized as safe) attributes [25,26,27,28]. The synthetic *alaD* genetic construct was designed insilico, synthesized and expressed into native *P. acidilactici* BD16 strain using recombinant shuttle vector pLES003*alaD*. L-alanine production in the recombinant strain was achieved using fed-batch fermentation by supplementing dextrose after defined intervals under oxygen deprived conditions to achieve enhanced production titers. Further, the produced L-alanine was analyzed for its enantiomeric purity, spectral and chromatographic attributes and crystal morphology using various analytical techniques.

## 2. Materials and Methods

### 2.1. Microorganisms and Growth Conditions

Native *P. acidilactici* BD16 MTCC 10973 and its recombinant were revived in De Man Rogosa and Sharpe medium (MRS), containing dextrose (20 g/L), beef extract (10 g/L), peptone (10 g/L), sodium acetate (5 g/L), yeast extract (5 g/L), triammonium citrate (2 g/L), dipotassium hydrogen phosphate (2 g/L), magnesium sulfate (0.1 g/L), manganous sulfate (0.05 g/L) and Tween-80 (1 mL/L), pH of 6.5 ± 0.2 under microaerophilic and stationary conditions at 37 °C for 24 h. MRS broth was supplemented with 25 µg/mL of erythromycin for the screening, selection and further culturing of the recombinant strains. 

### 2.2. Method of Culturing of Host Strain under Microaerophilic Conditions

Recombinant cell cultures were prepared by adding 2% *v*/*v* freshly grown inoculum (containing 10^6^ cfu/mL) in 25 mL test tubes containing 18–20 mL of sterile MRS media supplemented with 25 µg/mL of erythromycin, followed by incubation at 37 °C for 24 h under stationary conditions. Use of higher culture volumes in test tubes facilitated the development of oxygen depression conditions.

### 2.3. Designing of Synthetic Alanine Dehydrogenase (alaD) Gene Cassette

**Figure 1:**
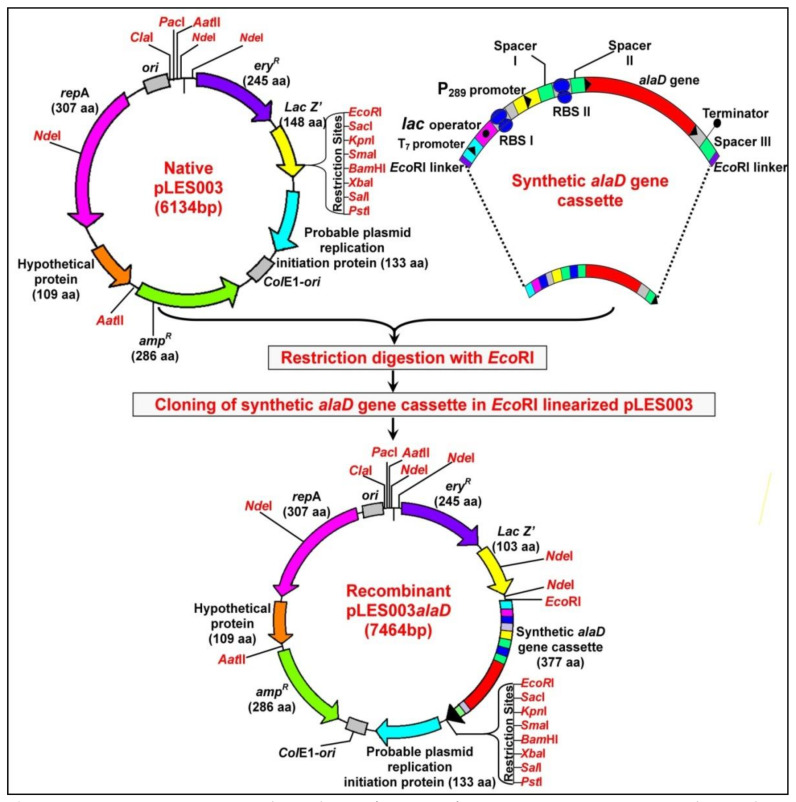
Genetic, restriction and regulatory features of native pLES003 vector (6134bp) and construction of recombinant pLES003*alaD* vector (7464 bp) expressing synthetic *alaD* gene cassette.

A synthetic alanine dehydrogenase gene (*alaD*) cassette was designed insilico by the application of online computer-assisted protocols using the reference *alaD* gene sequence of *Geobacillus stearothermophillus* vide GENBANK accession no. 061581031.1 [11,25]. Sequence manipulation suite 2 tools were used for synthetic gene designing and to overcome any possibility of codon biasing in the heterologous *P. acidilactici*. In order to enhance gene expression in this heterologous host, an auto-inducible Pediococcal promoter P_289_ (GENBANK accession no. GQ214404) and its essential control elements, such as RBS, were integrated upstream to the synthetic *alaD* gene cassette. In addition, *EcoR1* linkers were attached to the terminal flanks of the insilico-designed synthetic construct. Further, the designed genetic construct was sent to GenScript Private Limited, USA, for synthesis. Synthetic *alaD* gene (1336 bp) was obtained as a cloned construct in the pUC57 vector. The sequence of the whole synthetic *alaD* gene cassette was submitted to the GENBANK database under the accession number MT108231. The detailed genetic, restriction and regulatory features of the synthetic *alaD* gene cassette are depicted in Figure 1.

### 2.4. Subcloning of Synthetic alaD Gene Cassette and Construction of Recombinant Plasmid pLES003alaD

The synthetic *alaD* gene cassette (1336 bp) was further subcloned into pLES003 (provided by Dr. Masafumi Noda, Associate Professor, Graduate School of Biomedical and Health Sciences, Hiroshima University, Japan). The pLES003 vector is a shuttle vector that can replicate in both Gram-negative *E. coli* and Gram-positive lactic acid bacteria [29]. Its use has already been reported to enhance the phenolic biotransformations and vanillin production in *P. acidilactici* BD16 [25]. The recombinant pLES003*alaD* was constructed by *EcoRI* restriction digestion of the recombinant pUC57 vector (using 1 IU enzyme per 100 ng of plasmid) in a water bath at 37 °C for 40 min, followed by subcloning of the 1336 bp long *alaD* synthetic gene cassette into an *EcoRI* linearized vector pLES003 using T4 DNA ligase (1 IU enzyme per 100 ng of plasmid) at 25 °C overnight. Size confirmation of the restricted fragments and recombinant pLES003*alaD* was carried out by performing agarose gel electrophoresis (using 1% *w*/*v* agarose gels) and after visualizing under UV transilluminator. 

### 2.5. Preparation of the Competent P. acidilactici BD16 and Its Transformation by CaCl_2_-Heat Shock Method

A native *P. acidilactici* BD16 culture was grown in MRS broth and incubated at 37 °C for 24 h. After three subculturings, the freshly grown bacterial culture (2% *v*/*v* containing 10^6^ cfu/mL) was taken as inoculum and transferred into 100 mL of sterile MRS broth (containing 3% glycine and 50 mM D,L-threonine) and kept at 37 °C for about 24 h. Cells were then pre-chilled in a nice bath for about 15 min and later harvested by centrifuging 5–10 mL of culture at 5000 rpm at 4 °C for 10 min. The pellet was then washed twice using a wash solution (containing 0.5 M sucrose and 10% *v*/*v* glycerol) and suspended in 1 mL of buffer solution containing 600 mM sucrose, 1 mM K₂HPO_4_ and 1 mM MgCl₂, pH of 7 for about 30 min. For the preparation of competent cells, the bacterial suspension was centrifuged at 5000 rpm for 5 min, resuspended in 1 mL of CaCl₂ containing 15% *v*/*v* glycerol and stored at −80 °C for further use [25].

After thawing at room temperature, 100 µL of the competent cell preparation was mixed with 10 µL of recombinant plasmid pLES003*alaD* and incubated for 30 min in the ice bath. It was followed by a 2 min heat shock at 45 °C in a water bath to facilitate the genetic transformation. About 1 mL of the outgrowth medium (MRS broth containing 0.5 M sucrose, 20 mM MgCl₂ and 2 mM CaCl₂) was added to the cells for their immediate revival and kept at 37 °C for 1 h. After genetic transformation, recombinant cells were screened and selected on MRS agar plates containing 25 μg/mL of erythromycin [25].

### 2.6. Determination of Transformation Efficiency, Plasmid Segregational Stability and Copy Number of the Recombinant pLES003alaD Vector 

Plasmid segregational stability was determined in terms of capability of the recombinant bacterial strain to maintain recombinant plasmid after growth for 10–100 generations in the presence of selection pressure. Subculturings were performed on a regular basis in MRS medium containing 25 μg/mL of erythromycin. Plasmid stability was analyzed for 100 consecutive subculturings at 37 °C for 24 h by extracting the recombinant plasmid pLES003*alaD* from the culture broth after regular intervals and further their identification on 1% *w*/*v* agarose gels with respect to the DNA ladder (examined under UV-transilluminator). 

The plasmid copy number (PCN) indicates the copy number of the recombinant plasmid present per chromosome in a desired bacterial strain. The PCN value of the recombinant plasmid was determined using the Avogadro number method and the transformation efficiency was calculated using the method described previously by Kaur and coworkers [27].

### 2.7. Fed-Batch Fermentation for Production of L-Alanine

L-alanine production in the recombinant *P. acidilactici* BD16 (*alaD*^+^) was carried out using fed-batch fermentation at a flask level under oxygen deprived conditions. Minimal salt medium (MSM), containing sodium acetate (5 g/L), tri-ammonium citrate (2 g/L), magnesium sulfate (0.1 g/L), manganese sulfate (0.05 g/L), di-potassium hydrogen phosphate (2 g/L), erythromycin (20 μg/mL) and Tween-80 (1 mL/L), pH of 6.5, was used as a basal medium for conducting the experimental trials. Before initiating the fed-batch fermentation, different carbon sources, viz., glucose, dextrose, fructose, maltose, mannitol and sucrose (at a concentration of 20 g/L), were screened to evaluate carbon preference of the recombinant cell culture. Selected carbon source—dextrose (40 g/L; 200 mM)—was then fed into the recombinant culture broth repeatedly after every 6 h intervals. A higher volume of media was added to the flasks for maintaining oxygen deprived conditions (250 mL Erlenmeyer flask containing about 200 mL of MSM). Fed-batch fermentation was carried out for 42 h at 37 °C and L-alanine concentration in the recombinant culture broth was estimated after every 2 h till 36 h, as per the standard spectrophotometric method described by Shah and coworkers [30]. Sugar estimation was carried out using the DNS method after every 2 h intervals [31]. All the estimations were performed in triplicate and values are presented as the mean of the triplicate observations.

### 2.8. Quantitative Estimation of L-Alanine Production in Recombinant P. acidilactici BD16 (alaD^+^)

Concentration of L-alanine produced by the recombinant cell culture was estimated spectrophotometrically using a standard procedure [30]. For estimation, 1 mL of freshly grown recombinant culture was taken and centrifuged at 5000 rpm for 5 min to obtain a cell-free supernatant (CFS). To the CFS, 5 mL of Dichlone reagent (62.5 mg of Dichlone dissolved in dimethyl sulphoxide), was added and kept for incubation in the boiling water bath for 10 min to allow development of the orange color. The reaction mixture was allowed to cool down and was further diluted in 94 mL (0.5 M) of HCl. Absorbance was taken at 470 nm against blank lacking crude enzyme (CFS) and L-alanine concentration was calculated using standard curve. All the estimations were performed in triplicate and data are presented as mean of all the three values. 

### 2.9. Extraction of L-Alanine from Recombinant Culture Broth by Crystallization

After optimization, L-alanine produced by the recombinant *P. acidilactici* BD16 (*alaD*^+^) cultures was extracted from the 24 h old fermentation broth using the crystallization method as described by Shibatani and co-workers [10] with few modifications The 24 h old fermentation broth was centrifuged at 5000 rpm for 5 min to obtain CFS. To the CFS (100 mL), 3 g of activated charcoal was added and the pH of the reaction mixture was adjusted to 4.0 prior to boiling treatment. The reaction mixture was allowed to boil and then filtered at temperature >60 °C using a Whatman filter paper No. 1. An equal volume of methanol was added to the filtrate and kept at 4 °C for 2–3 days to facilitate the crystallization process. Precipitated crystals of L-alanine were separated from the filtrate by passing through Whatman’s filter paper and further recrystallized using aqueous methanol. Crystals were then dried completely by vacuum desiccation and weighed for estimating L-alanine yield.

### 2.10. Analytical and Morphological Characterization of L-Alanine Crystals

Analytical characterization for the confirmation of L-alanine crystals was carried out by Fourier transform infrared spectroscopy (FTIR) and HPLC methods. FTIR analysis was carried out using a Perkin Elmer FTIR spectrometer (Model no. RX-1 FT-IR systems, Japan) equipped with a KBr beam splitter. Crystals were mixed with dried KBr and provided with a pressure of 5 × 10^6^ Pa to create a clear pellet with a 13 mm diameter and 1 mm thickness. Then, the FTIR spectra of the standard and microbial L-alanine samples were compared in the spectral range of 3500–500 cm^−1^ [32].

For HPLC analysis, an RP-HPLC instrument, equipped with a PDA detector (SPD-M20A, Shimadzu, Japan), an auto-sampler, Nucleodur, C18 column (4.6 × 250 mm, 5 μm particle size) and the LC-solution software, was used. A gradient elution was performed with solvent A (10 mM sodium acetate in 5% acetonitrile, pH of 6.3) and B (Acetonitrile) with a flow rate of 1 mL/min at 30 °C. A volume of 100 µg/mL of the extracted L-alanine and standard L-alanine were used for the comparison. For derivatization, sample and standard solutions were added to the vials containing Dansyl chloride (20 mg/mL) prepared in acetonitrile. Vials containing reaction mixtures were then incubated in a water bath at 70 °C for 1 h. Reaction mixtures were then allowed to cool down at room temperature, followed by filtration using 0.45 μm polytetrafluoroethylene filters. Samples were then injected into the HPLC system and quantification was performed at 250 nm. The spectral peaks obtained at a specific retention time in the case of standard L-alanine and microbial L-alanine produced were later compared for confirmation purposes [33].

Morphological characterization of L-alanine crystals was also performed by scanning electron microscopy (SEM; Jeol JSL-6510LV). For SEM analysis, a very thin layer of gold and platinum was coated on the surface of the crystals to initiate the electrical conductance and for reducing the dry charging effects. Crystal morphology was then studied at specific conditions of accelerating voltage and magnifications [32].

### 2.11. Enantiomeric Purity of L-Alanine Produced by Recombinant P. acidilactici BD16 (alaD^+^)

The enantiomeric purity of the microbial L-alanine was evaluated by analyzing its specific optical rotation using a polarimeter. The polarographic conditions used were c=10, 6 N HCl. The specific rotation of the crystallized L-alanine obtained from the fermented broth was compared with the specific rotation of the standard L-alanine. Further, the optical purity (enantiomeric excess) of the L-isomer of alanine was calculated using the standard formula,

% Optical purity of sample = 100 × (specific rotation of the sample)/(specific rotation of a pure enantiomer).(1)

### 2.12. Statistical Analysis

All the estimations were performed in triplicate and values were averaged to calculate the values of mean, standard deviation and standard error using the MS Excel software to authenticate statistical significance of the observed data.

## 3. Results

### 3.1. Designing of Synthetic alaD Gene Construct and Construction of Recombinant pLES003alaD Vector

**Figure 2:**
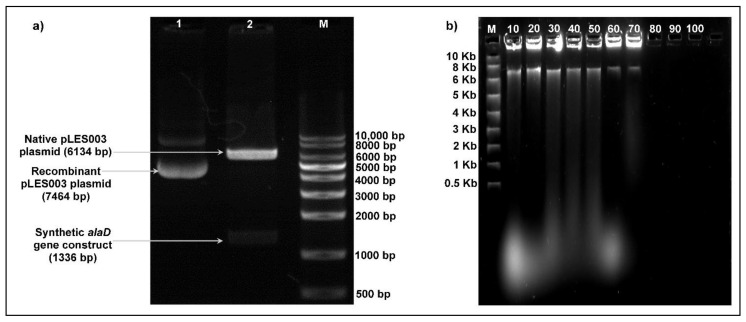
(**a**) Gel analysis of the recombinant pLES003*alaD* vector, where Lane 1 shows the recombinant pLES003*alaD* vector, Lane 2 shows the *EcoRI* linearized recombinant pLES003*alaD* and Lane M shows the DNA ladder. (**b**) Segregational stability of the recombinant pLES003*alaD* (7464 bp) analyzed on 1% *w*/*v* agarose gels, where Lane M shows the DNA ladder and Lanes 10–100 show the number of subculturings for which the stability of the recombinant plasmid was studied.

The synthetic *alaD* gene construct designed using online computational tools was obtained as a cloned construct in the pUC57 vector from GenScript, USA. The synthetic *alaD* gene cassette of 1336 bp was extracted from the recombinant pUC57 vector by EcoRI digestion and further ligated into the EcoRI linearized pLES003 plasmid (6134 bp) to generate a recombinant pLES003*alaD* vector (7464 bp) (Figure 1). Agarose gel electrophoresis was performed for size confirmation of the DNA constructs, which were visualized under UV illumination at 312 nm (Figure 2a).

### 3.2. Transformation Efficiency, Plasmid Copy Number and Segregational Stability of Recombinant pLES003alaD Vector

The shuttle vector pLES003 is a low copy number plasmid constructed for obtaining gene expression lactic acid bacterial strains [29,34]. However, the recombinant plasmid pLES003*alaD* showed a PCN value of ~10 copies per cell in the *P. acidilactici* BD16 (*alaD*^+^), with a transformation efficiency of 8 × 10^6^ cfu/mL. The recombinant plasmid also displayed an interesting segregational stability up to 70 subculturings in the recombinant host strain (Figure 2b), after which plasmid loss was evidenced in the selected expression host.

### 3.3. L-Alanine Production in Recombinant P. acidilactici BD16 (alaD^+^) Using Fed-Batch Fermentation

**Figure 3:**
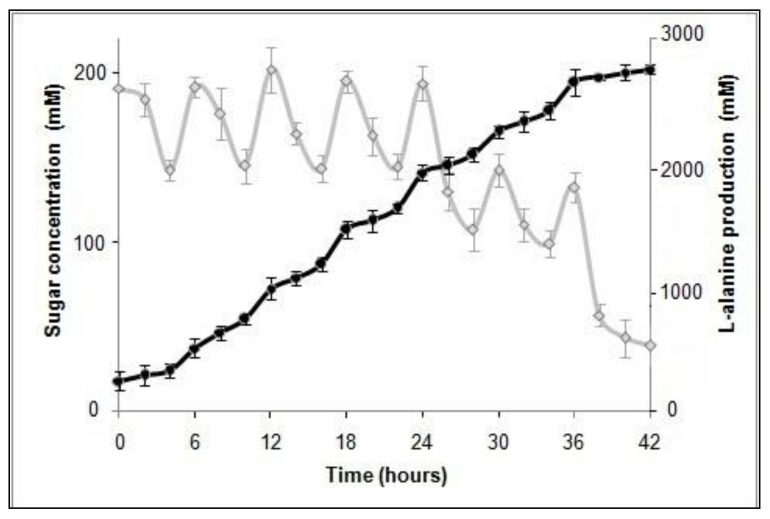
Fed-batch fermentation and production of L-alanine in the recombinant *P. acidilactici* BD16 (*alaD*^+^).

Dextrose was added to the fed-batch fermentation system after every 6 h till 36 h post inoculation. Supplementation of dextrose at defined intervals enhanced L-alanine production levels significantly upto 229.12 g/L, 2572 mM (which counts for 91.8% of the theoretical yield) after 42 h under oxygen deprived conditions (Figure 3). The L-alanine titers obtained in the present study are quite impressive, compared to most of the previous reports on L-alanine production using recombinant or wild type microorganisms (Table 1). L-alanine production in the wild type *P. acidilactici* BD16 strain (lacking the recombinant plasmid pLES003*alaD*) was also quantified, which was almost negligible (4.71 mM) to report here in comparison to the developed recombinant strain (2572 mM), when estimated spectrophotometrically.

### 3.4. Crystallization and Enantiomeric Purity of Heterologously Produced L-Alanine

The use of an auto-inducible promoter P_289_ for the expression of the synthetic *alaD* gene construct resulted in the over-expression of the recombinant AlaDH enzyme (12.44 IU/mL, using 55 mM pyruvate as an inducer—unpublished data) and corresponding higher L-alanine titers in the recombinant culture broth, as evidenced by appearance of the turbidity in the recombinant culture broth after 18 h of incubation. The accumulation of L-alanine beyond its solubility triggered the formation of L-alanine crystals. Further, to recover soluble L-alanine from the culture broth, crystallization was performed. After crystallization, the crystal L-alanine titer was quantified, which is about 95% of the total yield (217.54 g/L). The amount was a little lower than the actual L-alanine titer quantified via spectrophotometric assay, possibly due to the inevitable losses observed during crystallization. The dried crystals were used to further study enantiomeric purity of L-alanine. Using a polarimeter, specific rotations of standard and test samples were calculated as +14.2° and +13.8°. Positive values of the observed specific rotations correspond to the L-enantiomers (Pubchem Database). From the specific rotation, optical purity in terms of enantiomeric excess was calculated as 97%, which signifies 97% purity of the L-isomer of alanine produced by the recombinant strain. Earlier, Shibatani and co-workers [10] also reported the optical purity of L-alanine using a polarimeter under similar conditions (c = 4, 6 N HCL) in the form of positive specific rotation values (+14.3°) which corresponded to 93% purity of the L-isomer of alanine.

### 3.5. Morphological and Analytical Characterization of L-Alanine Crystals 

**Figure 4:**
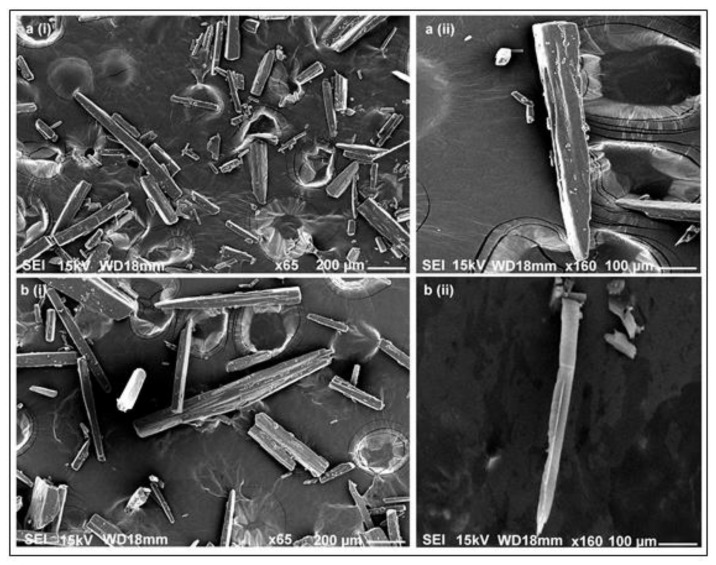
Scanning electron microscopic images of pure L-alanine crystals taken at (**a**) (i) ×65 and (**a**) (ii) ×160 resolution and of L-alanine produced by the recombinant strain taken at (**b**) (i) ×65 and (**b**) (ii) ×160 resolution, respectively, both showing needle-like crystal morphology.

L-alanine crystals were characterized using bioanalytical techniques such as FTIR, HPLC and SEM. The morphology of L-alanine crystals obtained from the fermentation broth was compared with the properties of commercially available L-alanine crystals (Himedia) by SEM. SEM imaging revealed a high similarity between the standard and microbial L-alanine crystals at different resolutions (Figure 4). Figure 4a (i) and (ii) of the standard L-alanine crystals (at a resolution of ×65 and ×160, respectively) depict a needle-like morphology. L-alanine produced by the recombinant strain also showed a similar needle-like morphology at the respective resolutions (at ×65 and ×160), as depicted in Figure 4b (i) and (ii). A similar morphology of L-alanine crystals has also been reported widely in the previous literature [10,37]; thus, it confirms the presence of L-alanine in the crystals obtained from the fermentation broth.

**Figure 5:**
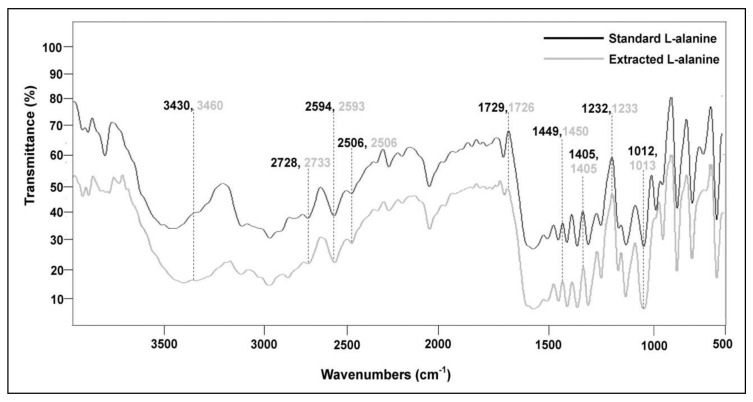
A close similarity in the FTIR spectra of standard L-alanine and L-alanine extracted from the recombinant *P. acidilactici* BD16 (*alaD^+^*).

FTIR spectra of both standard L-alanine and L-alanine extracted from the recombinant fermentation broth were generated to identify similarities in their relevant functional groups. As evident from Figure 5, the FT-IR spectrum of the microbial L-alanine is highly consistent with that of the standard L-alanine in the region of 3500–500 cm^−1^. The broad absorption bands at 3430 and 3460 cm^−1^ in the spectra were associated with specific N-H stretching of the primary aliphatic amines. C-H stretching of the methyl group was confirmed by the presence of bands at 2728 and 2733 cm^−1^, respectively, and C=O stretching of the carboxylic group was confirmed by the presence of absorption bands at 1729 and 1726 cm^−1^. Typical curved absorption bands at 2594, 2593 and 2506 cm^−1^ were attributed to a typical O-H stretching and bands at 1449, 1450 and 1405 cm^−1^ to a typical O-H bending of the carboxylic group. The sharp signals at 1232, 1233, 1012 and 1013 were credited to C-N stretching of the primary amines [38,39,40].

**Figure 6:**
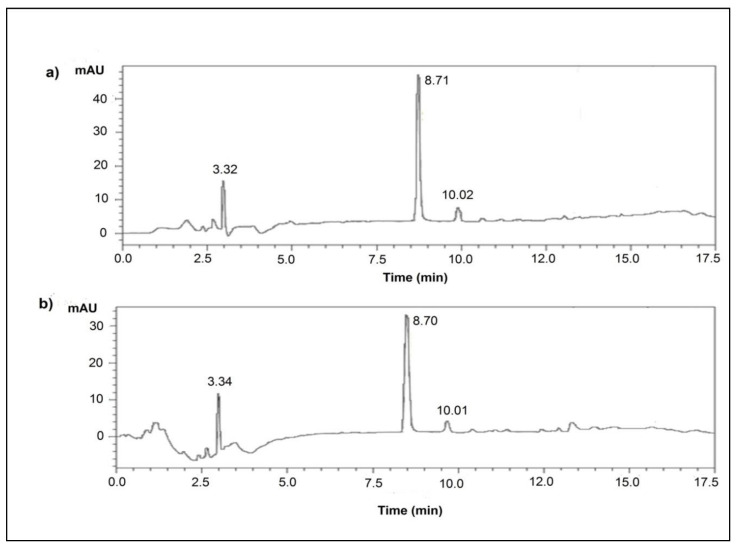
RP-HPLC-PDA chromatograms of the aqueous samples, where (**a**) is standard L-alanine and (**b**) is L-alanine produced by the recombinant strain. Analysis was performed using a C_18_ column (250 × 4.6 mm) with a column particle size of 10 µm, mobile phase (sodium acetate and acetonitrile) at a flow rate of 1.0 mL/min, a detection wavelength of λ = 250 nm and a 10 µL injection volume. Presence of a sharp peak at a retention time of 8.7 min in the (**a**) standard chromatogram and (**b**) chromatogram of the test sample confirms the presence of L-alanine in the crystals, wherein the minor peaks at RTs 3.3 and 10.0 min may signify the contaminant amino acids, such as L-glutamic acid and L-phenylalanine, respectively.

Analytical characterization and further confirmation of the L-isomer of alanine in the extracted crystals was also carried out by HPLC analysis. HPLC chromatograms of both the standard and extracted L-alanine were acquired under optimized conditions for comparative analysis. Presence of sharp and intense peak at a retention time of 8.7 min in both the chromatograms revealed and confirmed the presence of L-alanine (Figure 6).

## 4. Discussion

A plethora of microbial strains have been harnessed for their capacity to produce L-alanine at the laboratory scale (Table 1). In the case of wild type microbial strains, such as *Micrococcus* sp., *Pyrococcus furiosus*, *Salmonella typhimurium*, etc., limited production titers and poor racemic resolution were observed [41,42]. Later on, with the development of genetically modified strains, further enhancement of the fermentative production titers of L-alanine was observed. However, L-alanine production using recombinant strains remained challenging due to the requirement of long fermentation hours, lesser stability, poor yield, less purity and co-product formation. This has led to the implementation of advanced strain improvement approaches, such as metabolic engineering, for the development of highly stable and robust L-alanine producing microbial strains capable of withstanding the fermentation pressures of large scale industrial fermenters. 

The concept of metabolic engineering for L-alanine production was first adopted by Uhlenbusch and co-workers, who engineered a Gram-negative, ethanologenic bacterium *Zymomonas mobilis* by expressing the *Bacillus sphaericus alaD* gene. Despite high intracellular L-alanine levels of 23.1 g/L (260 mM) at the beginning of the excretion phase, a subsequent decrease in the alanine yield (7.5 g/L, 80–90 mM after 24 h) was observed along with co-product formation. This might be due to the end product degradation or because of the fact that the central metabolism of *Zymomonas mobilis* is naturally optimized for directing the glycolytic flux towards ethanol due to translational competition [18]. A homolactic *L. lactis* strain was converted into homo-alanine by the disruption of the L-lactate dehydrogenase gene (*ldh*) along with the overexpression of the *B. sphaericus alaD* gene under the control of the nisA promoter. However, only 13 g/L (146 mM) of D,L-alanine was produced from 18 g/L of glucose [2]. This study also highlights the extensive role of codon biasing and presence of highly active competing pathways in the heterologous host.

Further, a strain of *E. coli* was genetically engineered using the pTrc99A-*alaD* plasmid for the heterologous expression of *B. sphaericus alaD* under the control of a strong promoter. A two-phase fermentation process, codon biasing, translational competition between the lactate dehydrogenase and AlaDH enzymes for pyruvate could have led to low alanine titers of 32 g/L (359 mM) in 27 h [3] Then, in 2006, Smith and coworkers engineered an *E. coli* strain using extensive gene knockouts and reported an 88 g/L (988 mM) alanine production in 48 h. The use of multicopy plasmids, undoubtedly, has raised the fermentation productivity of L-alanine, but, at the same time, the nutritionally complex media formulation has aroused a need of multistage fermentation process and racemic bioconversion [36].

Further, in 2007, an *E. coli* strain was metabolically engineered with the integration of the *G. stearothermophillus alaD* gene and knockout of competing pathways genes, such as *pflB*, *ackA*, *adhE*, *ldhA*, *mgsA* and *dadX*. The recombinant *E. coli* strain produced 114 g/L (1280 mM) of L-alanine from 120 g/L of glucose in 48 h under oxygen limiting conditions [11]. Further, Jojima and coworkers, in 2010, genetically engineered *C. glutamicum* by inactivation of the genes associated with organic acids production and over-expression of the *Lysinibacillus sphaericus alaD* gene for enhancing sugar metabolism under oxygen deprivation conditions. The resulting strain produced only 98 g/L (1097 mM) of L-alanine using fed-batch fermentation after 32 h, probably due to the retarded glucose consumption [15]. Later, in 2012, researchers of the same group reported the highest production of L-alanine by stepwise over-expression and chromosomal integration of four glycolytic genes (*gapA*, *pyk*, *pfk* and *gpi*) in *C. glutamicum* having heterologously expressed the *alaD* gene of *L. sphaericus*. Over-expression of glycolytic genes reduced the intracellular NADH/NAD+ ratio, which eventually led to an improvement in the glucose consumption, thus hastening the overall alanine productivity under oxygen deprivation conditions. The metabolically engineered strain thus produced 216 g/L (2430 mM) of L-alanine in 48 h using fed-batch fermentation, which was 6.4-fold higher than the wild type strain [16]. 

Further, using a thermo-regulated genetic switch, a high-level L-alanine production was achieved in the *E. coli* derivative expressing *G. stearothermophillus alaD* gene. The resulting strain produced L-alanine titers of 120.8 g/L (1347 mM) in 24 h under oxygen limiting conditions at 42 °C [17]. However, two-phase fermentation systems and heat transfer complications have limited the use of recombinant strain for industrial level production of L-alanine. Thus, to conclude, the complex genetic manipulations, codon biasing, fastidious nutritional requirements, translational competition between sister pathway enzymes leading to undesirable side reactions and co-product formation, heat generation, end product inhibition and requirements for the two-stage fermentation process and chiral separation have limited their utilization for industrial level production of L-alanine.

Therefore, in the present study, *P. acidilactici* BD16, a microaerophilic strain, was chosen as a heterologous host for the production of L-alanine under anoxic conditions. Oxygen deprived conditions were found to be favorable for the high L-alanine titers observed in various recombinant strains [11,15,16,17]. Further, the use of the synthetic *alaD* gene with *Pediococcus acidilactici* optimized codons and a moderately high copy number shuttle vector pLES003*alaD* with cloned auto-inducible P_289_ promoter led to the constitutive expression of the AlaDH enzyme in the recombinant host, thus yielding a very high L-alanine production titer. The study provides a simple, one-stage fed-batch fermentation process for efficient L-alanine production in the heterologous host without hampering its natural metabolism and cell viability. The novelty of the study is the use of a synthetic systems biology-mediated metabolic engineering strategy that abolished the need of complex gene knock-outs for engineering a microbial strain, which may hinder maximal expression of the desired cloned gene. The robust expression system, ideal GRAS host, simple media formulation and a superlative fermentation yield in the fed-batch process validate the significance of the present study.

However, in the future, the authors would like to work either on the segregation stability of the recombinant plasmid or on the use of gene integration approaches for achieving very high fermentation titers of L-alanine in the recombinant *P. acidilactici* BD16. Statistical optimizations and scale-up studies at the fermenter level could also be conducted before proposing a commercial scale L-alanine biosynthetic process. Potential applications of the recombinant *P. acidilactici* BD16 (*alaD*^+^) strain for the insitu L-alanine production in various foods, beverages and pharmaceuticals could also be explored further.

## Figures and Tables

**Table 1:** Comparative account of L-alanine production using recombinant microbial strains.

Microorganism	Genetic Modifications	Growth and Fermentation Conditions	Fermentation Process	Time (h)	L-Alanine Production in g/L (mM)	Enantiomeric Purity (%)	Reference
***Zymomonas mobilis*** **CP4(pZY73)** (Gram –ve)	*Bacillus sphaericus* IFO3525 *alaD*	Mineral salts medium containing 280 mM glucose, 85 mM ammonium sulfate; under anaerobic conditions	Batch	26	7.5 (84 mM)	Not reported	[18]
***Corynebacterium*** ***glutamicum* AL107** **(pOBP107)** (Gram +ve)	*Arthrobacter oxydans* HAP-1 *alaD*	Corn steep liquor medium containing 1110 mM glucose, 44.8 mM *DL*-alanine; limited oxygen conditions	Batch	70	71 (797 mM)	>99	[35]
***E coli* AL1** **(pOBP1)** (Gram –ve)	*A. oxydans* HAP-1 *alaD*	Mineral salt medium containing 110 mM glucose; limited oxygen conditions	Batch	40	8 (90 mM)	Not reported	[35]
***Arthrobacter*** ***oxydans*** **DAN 75** (Gram +ve)	Alanine racemacedeficient	Mineral salt medium containing 832.6 mM glucose, 2.24 mM *D*-alanine; shaking conditions	Two-stage fed-batch	120	77 (864 mM)	98	[12]
***Lactococcus*** ***lactis* NZ3950** **(pNZ2650)** (Gram +ve)	*B. sphaericus* IFO3525 *alaD,* Δ*ldhA*	M17 rich medium containing 100 mM glucose, 2.25 mM D-alanine, 150 mM Ammonium sulfate; shaking conditions at 120rpm	Batch	17	13 (146 mM)	85–90	[2]
***L. lactis* PH3950** **(pNZ2650)** (Gram +ve)	*B. sphaericus* IFO3525 *alaD,* Δ*ldhA*, Δ*alr*	M17 rich medium containing 100 mM glucose, 2.25 mM D-alanine; 150 mM Ammonium sulfate; shaking conditions at 120rpm	Batch	17	Not reported	>99	[2]
***E. coli* ALS887** **(pTrc99A*-alaD)*** (Gram –ve)	*B. sphaericus* *IFO3525 alaD,* Δ*ldhA*, Δ*aceF*	Medium containing 666 mM glucose, 1261 mM ammonium chloride; oxygen limited conditions	Two-stage, fed-batch	27	32 (359 mM)	Not reported	[3]
***E. coli* ALS929** **(pTrc99A*-alaD)*** (Gram –ve)	*B. sphaericus* IFO3525 *alaD,* Δ*pfl*, Δ*pps*, Δ*poxB*, Δ*ldhA*, Δ*aceF*	Medium containing 999 mM glucose; oxygen limited conditions	Two-stage fed-batch	23 h anaerobic phase (48 h total fermentation time)	88 (988 mM)	Not reported	[36]
***E. coli*XZ132** (Gram –ve)	*Geobacillus* *stearothermophilus alaD*, Δ*pfl*, Δ*ackA*, Δ*adhE*, Δ*ldhA*, Δ*mgsA*, Δ*dadX*	Low salt medium containing 666 mM glucose; anaerobic conditions	Batch	48	114 (1279 mM)	>99	[11]
***C. glutamicum*** (Gram +ve)	*Lysinibacillus sphaericus alaD, gapA*, Δ*ldhA*, Δ*ppc*, Δ*alr*	Minimal salts medium containing 888 mM glucose, 52.97 mM ammonium sulfate; Limited oxygen conditions	Fed-batch	32	98 (1097 mM)	99.5%	[15]
***C. glutamicum*** **GLY3/pCRD500** (Gram +ve)	*L. sphaericus alaD,* Δ*ldhA*, Δ*ppc*; stepwise over-expression and chromosomal integration of four glycolytic enzymes encoded by *gapA*, *pyk, pfk*, *gpi* genes	BT medium containing 1600 mM glucose; with agitation but without aeration.	Two stage fed-batch	48	216 (2430 mM)	-	[16]
***C. glutamicum*** **GLY3/pCRD914** (Gram +ve)	*L. sphaericus alaD*	BT medium containing 1600 mM glucose; with agitation but without aeration.	Two stage fed-batch	72	275 (3090 mM)	-	[16]
***E. coli*** **B0016-060BC** (Gram –ve)	*G. stearothermophilus alaD*, *∆ack, ∆pta,* *∆pflB, ∆adhE, ∆frdA, ∆ldhA ∆alr*	M9-1 medium containing 2837 mM glucose; oxygen limited phase at 42 °C	Two stage fed-batch	24	120.8 (1347 mM)	-	[17]
***P. acidilactici******BD16*** (Gram +ve) **(Wild type)**	No genetic manipulation	MRS medium containing 100 mM dextrose; under microaerophilic and stationary conditions	Batch	24	0.42 (4.71 mM)	-	Present study
***P. acidilactici* BD16** (Gram +ve) **(Codon optimized)**	pLES003 containing synthetic *alaD* gene cassette	Minimal Salt Medium containing 1400 mM dextrose, 8.22 mM tri-ammonium citrate; oxygen limited conditions	Fed-batch	42	217.54(2442mM)	97%	Present study

Where *alaD*, alanine dehydrogenase gene; *aceF*, gene encoding one of the proteins of pyruvate dehydrogenase complex; *ackA*, acetate kinase; *adhE*, alcohol/aldehyde dehydrogenase; *alr*, alanine racemase; *gapA*, glyceraldehyde-3-phosphate dehydrogenase; *gpi*, glucose-6-phosphate isomerase; *ldh*, lactate dehydrogenase; *msgA*, methylglyoxal synthase; *pfl*, pyruvate formate lyase; *ppc*, phosphenol pyruvate decarboxylase; *pps*, phosphenolpyruvate synthase; *pox*, pyruvate oxidase; *pyk*, pyruvate kinase; *pfk*, phosphofructokinase.

## Data Availability

The data generated from the study is clearly presented and discussed in the manuscript.
